# Allergen immunotherapy for IgE-mediated food allergy: protocol for a systematic review

**DOI:** 10.1186/s13601-016-0113-z

**Published:** 2016-07-05

**Authors:** Sangeeta Dhami, Ulugbek Nurmatov, Giovanni Battista Pajno, Montserrat Fernandez-Rivas, Antonella Muraro, Graham Roberts, Cezmi Akdis, Montserrat Alvaro-Lozano, Kirsten Beyer, Carsten Bindslev-Jensen, Wesley Burks, George du Toit, Motohiro Ebisawa, Philippe Eigenmann, Edward Knol, Mika Makela, Kari Christine Nadeau, Liam O’Mahony, Nikolaos Papadopoulos, Lars Poulsen, Cansin Sackesen, Hugh Sampson, Alexandra Santos, Ronald van Ree, Frans Timmermans, Aziz Sheikh

**Affiliations:** Evidence-Based Health Care Ltd, Edinburgh, UK; Division of Population Medicine Neuadd Meirionnydd, School of Medicine, Cardiff University, Heath Park, Cardiff, UK; Allergy Unit, Department of Pediatric, University of Messina, Messina, Italy; Allergy Department, Hospital Clínico San Carlos, IdISSC, Madrid, Spain; Food Allergy Referral Centre Veneto Region, Department of Women and Child Health, Padua General University Hospital, Padua, Italy; The David Hide Asthma and Allergy Research Centre, St Mary’s Hospital, Newport Isle of Wight, NIHR Respiratory Biomedical Research Unit, University Hospital Southampton NHS Foundation Trust, Southampton, UK; Faculty of Medicine, University of Southampton, Southampton, UK; Swiss Institute for Allergy and Asthma Research, Davos, Switzerland; Paediatric Allergy and Clinical Immunology Section, Hospital Sant Joan de Déu, Universitat de Barcelona, Barcelona, Spain; Pediatric Pneumology and Immunology, Charité Universitätsmedizin, Berlin, Germany; Icahn School of Medicine at Mount Sinai, New York, NY USA; Department of Dermatology and Allergy Centre, Odense University Hospital, Odense, Denmark; Department of Pediatrics, School of Medicine, University of North Carolina at Chapel Hill, Chapel Hill, NC USA; Division of Asthma, Allergy and Lung Biology, Department of Paediatric Allergy, MRC and Asthma Centre in Allergic Mechanisms of Asthma, King’s College London and St Thomas NHS Foundation Trust, London, UK; Department of Allergy, Clinical Research Center for Allergy and Rheumatology, Sagamihara National Hospital, Sagamihara, Kanagawa Japan; University Hospitals of Geneva and Medical School, University of Geneva, Geneva, Switzerland; Department of Dermatology and Allergology, University Medical Center Utrecht, Utrecht, The Netherlands; Department of Immunology, University Medical Center Utrecht, Utrecht, The Netherlands; Skin and Allergy Hospital, Helsinki University Hospital, Helsinki, Finland; Division of Immunology, Allergy and Rheumatology, Department of Pediatrics, Stanford University, Stanford, CA USA; Swiss Institute of Allergy and Asthma Research (SIAF), University of Zurich, Davos, Switzerland; Department of Allergy, 2nd Pediatric Clinic, University of Athens, Athens, Greece; Allergy Clinic, Copenhagen University Hospital, Gentofte, Denmark; Koç University Hospital, Istanbul, Turkey; World Allergy Organization (WAO), Milwaukee, WI USA; Division of Asthma Allergy and Lung Biology, Department of Paediatric Allergy, King’s College London/Guy’s, and St Thomas’ Hospital NHS Foundation Trust, London, UK; Department of Otorhinolaryngology, Academic Medical Center, Amsterdam, The Netherlands; Nederlands Anafylaxis Netwerk - European Anaphylaxis Taskforce, Dordrecht, The Netherlands; Allergy and Respiratory Research Group, The University of Edinburgh, Edinburgh, UK

**Keywords:** Allergy, Allergen immunotherapy, Food allergy, Therapy, Sensitisation

## Abstract

**Background:**

The European Academy of Allergy and Clinical Immunology (EAACI) is in the process of developing the EAACI Guidelines for Allergen Immunotherapy (AIT) for IgE-mediated food allergy. We seek to critically assess the effectiveness, cost-effectiveness and safety of AIT in IgE-mediated food allergy.

**Methods:**

We will undertake a systematic review, which will involve searching international biomedical databases for published, in progress and unpublished evidence. Studies will be independently screened against pre-defined eligibility criteria and critically appraised using established instruments. Data will be descriptively and, if possible and appropriate, quantitatively synthesised.

**Discussion:**

The findings from this review will be used to inform the development of recommendations for EAACI’s Guidelines on AIT.

**Electronic supplementary material:**

The online version of this article (doi:10.1186/s13601-016-0113-z) contains supplementary material, which is available to authorized users.

## Background

Food allergy is responsible for considerable morbidity and, in some cases, mortality [1]. Epidemiological studies have demonstrated that the prevalence and severity of food allergy may be increasing, particularly in children [2–5]. Food allergies can be divided into IgE-mediated acute allergic reactions manifesting as urticaria, vomiting, wheezing and anaphylaxis, and non-IgE-mediated food allergy which refers to delayed, cell-mediated reactions. This review is focused on IgE-mediated reactions.

Food allergies can be associated with significant reduction in quality of life, both of individuals who suffer from food allergy and their family members [6]. At present, avoidance measures are the cornerstone of management [7]. Difficulties in avoiding responsible food allergens can result in accidental exposure and the risk of triggering potentially life-threatening anaphylaxis [8]. Many individuals with food allergy therefore need to carry adrenaline (epinephrine) auto-injectors in order to self-manage anaphylaxis reactions. This approach is however perceived as restrictive and still leaves patients at risk if accidental exposure occurs.

Alternative approaches are therefore being investigated. In particular, there is considerable international interest in the role of immunotherapy, which involves repeated administration of very small, but gradually increasing doses of the antigens to which individuals are allergic in the hope of allowing safe exposure to the foods in question. Allergen immunotherapy (AIT) has, for example, over the last century become established clinical practice in relation to the treatment of severe pollen, insect venom and drug allergy [9]. However AIT has yet to become established in the routine management of food allergy.

The European Academy of Allergy and Clinical Immunology (EAACI) is in the process of developing the EAACI Guidelines for AIT, and this systematic review is one of five inter-linked evidence syntheses that are being undertaken in order to provide a state-of-the-art synopsis of the current evidence base in relation to evaluating AIT for the treatment of food allergy, allergic rhinoconjunctivitis, venom allergy and allergic asthma, and allergy prevention, which will be used to inform the formulation of key clinical recommendations. The focus of this review, which builds on our previous related reviews [10, 11], is on assessing the effectiveness, safety and cost-effectiveness of AIT in the management of IgE-mediated food allergy.

## Methods

### Search strategy

**Fig. 1:**
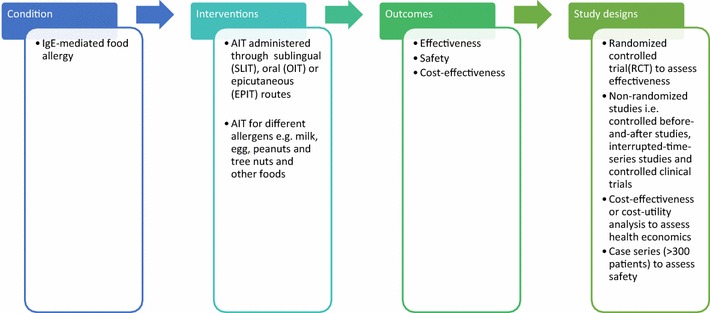
Conceptualization of systematic review of allergen immunotherapy for IgE mediated food allergy

A highly sensitive search strategy has been developed, and validated study design filters will be applied to retrieve articles pertaining to the use of AIT for IgE-mediated food allergy from electronic bibliographic databases. We have conceptualized the search to incorporate the four elements shown in Fig. 1.

To retrieve randomized controlled trials (RCTs), we will apply the Cochrane highly sensitive search strategy for identifying RCTs in MEDLINE [12]. To retrieve non-randomised studies, i.e. controlled clinical trials (CCT) and quasi-RCTs, we will use the Cochrane Effective Practice and Organisation of Care (EPOC) filter Version 2.4, available on request from the EPOC Group [13, 14]. To retrieve case series, we will use the filter developed by librarians at Clinical Evidence: http://clinicalevidence.bmj.com/x/set/static/ebm/learn/665076.html.

We will search the following databases:

- Cochrane Library including the:
  - CENTRAL (Trials)
  - Methods Studies
  - Health Technology Assessments (HTA)
  - Economic Evaluations Database (EED)
- MEDLINE (OVID)
- Embase (OVID)
- CINAHL (Ebscohost)
- ISI Web of Science (Thomson Web of Knowledge)
- TRIP Database (www.tripdatabase.com)
- Clinicaltrials.gov (NIH web).
- Current controlled trials (www.controlled-trials.com)
- Australian and New Zealand Clinical Trials Registry (http://www.anzctr.org.au)

The search strategy has been developed on OVID MEDLINE and then adapted for the other databases (see Additional file 1: Appendix). In all cases, the databases will be searched from inception to March 31, 2016. Additional references will be located through searching the references cited by the identified studies, and unpublished work and research in progress will be identified through discussion with experts in the field. We will invite experts who are active in the field from a range of disciplines and regions to add to the list of included studies by identifying additional published and unpublished papers and grey literature they are aware of and research in progress. We also, will search Web of Science to find published conference papers and all three major clinical trials repositories [Clinicaltrials.gov (NIH web); Current controlled trials (www.controlled-trials.com); Australian and New Zealand Clinical Trials Registry (http://www.anzctr.org.au) to identify trials in progress]. There will be no language restrictions employed; where possible, relevant literature will be translated into English.

### Inclusion criteria

#### Patient characteristics

We will focus on studies conducted on patients of any age with a physician confirmed diagnosis of IgE-mediated food allergy to milk, eggs, peanuts, tree nuts and other foods in which there is also confirmation of allergic status through positive skin prick tests, specific-IgE or food challenge tests.

#### Interventions of interest

This review is focused on AIT for different allergens, i.e. milk, eggs, tree nuts, peanuts and other foods, administered through the following routes: oral, sublingual and epicutaneous.

#### Comparators

We are interested in studies comparing food allergy AIT with placebo or routine care (i.e. adrenaline autoinjector with or without antihistamines) or no treatment.

#### Study designs

RCTs, will be used to investigate effectiveness (i.e. desensitization and tolerance) and impact on disease specific quality of life; health economic analysis will be used to assess cost-effectiveness; and RCTs and case series with a minimum of 300 patients will be used to assess safety. We will appraise the evidence by looking at higher levels of evidence such as individual RCTs. However, given the likelihood that we will find only a limited number of RCTs, we will also search for and include the following non-randomized studies (NRS): controlled before-and-after studies, interrupted-time-series studies and controlled clinical trials. Given the high inherent risk of bias in making inferences from such NRS, we will be very careful in making inferences from these data [15].

#### Study outcomes

##### Primary

- Desensitization (i.e. the ability to safely consume foods containing the allergen in question while on AIT) or tolerance (the ability to consume foods containing the allergen in question after discontinuing AIT) at food challenge, as defined in the relevant studies.
- Assessment of changes in disease specific quality of life using a validated instrument; in making this assessment we will focus on the minimal clinically important difference change in quality of life [16].

##### Secondary

- Safety as assessed by local and systemic reactions in accordance with the World Allergy Organization’s grading system of side-effects [17, 18].
- Health economic analysis from the perspective of the health system/payer as reported in studies.

### Exclusion criteria

The following exclusion criteria will be applied:

- Reviews, systematic reviews, discussion papers, non-research letters and editorials
- Animal studies
- Quantitative studies not employing systematic review or RCT, or employing NRS designs other than those detailed above
- Qualitative studies
- Case series (of <300 patients).

### Study selection

All references will be uploaded into the systematic review software Distiller and undergo initial deduplication. Study titles and abstracts will be independently checked by two reviewers according to the above selection criteria and categorized as: included, not included or unsure. Calibration will be undertaken after the first 50 screens to review any discrepancies between reviewers. For those papers in the unsure category, we will retrieve the full-text and re-categorize as above. Any discrepancies will be resolved through discussion and, if necessary, a third reviewer will be consulted. Full text copies of potentially relevant studies will be obtained and their eligibility for inclusion independently assessed. Studies that do not fulfil all of the inclusion criteria will be excluded.

### Quality assessment strategy

Quality assessments will independently be carried out on each study by two reviewers using the relevant quality assessment tools. Health economic studies will be assessed using the relevant CASP tool for economic evaluations [19]. RCTs, quasi-RCTs and CCTs will be assessed for generation of allocation sequence, concealment of allocation, baseline outcome measurements, baseline characteristics, incomplete outcome data, blinding of outcome assessor, protection against contamination, selective outcome reporting and other risks of bias. The Cochrane Risk of Bias tool will be used for RCTs and the Cochrane ACROBAT tool will be used for NRS [12]. Similarly, we will use the quality assessment form produced by the National Institute for Health and Clinical Excellence (NICE) to critically appraise case series [20]. Any discrepancies will be resolved by discussion or, if agreement cannot be reached, a third reviewer will arbitrate

### Data extraction, analysis and synthesis

Data will be independently extracted onto a customized data extraction sheet in Distiller by two reviewers, and any discrepancies will be resolved by discussion or, if agreement cannot be reached, by arbitration by a third reviewer.

A descriptive summary with summary data tables will be produced to summarize the literature. If clinically and statistically appropriate, meta-analysis will be undertaken using random-effects modeling given the known clinical heterogeneity between studies. In the event of finding significant statistical heterogeneity between studies (assessed using I^2^), this will initially be visually inspected and then, if appropriate, be investigated through the pre-specified subgroup and sensitivity analyses (see below). We will preferentially report on RRs with 95 % CIs A narrative synthesis of the data will also be undertaken.

### Sensitivity and subgroup analyses, and assessment for publication bias

Sensitivity analyses will be undertaken by comparing the findings between RCTs and NRS, and by comparing the results from studies that have employed double-blind placebo controlled food challenges versus those using other outcomes to assess for desensitization and/or tolerance.

Subgroup analyses will be undertaken to compare:

- Children (5–11 years) versus adolescents (12–17 years) versus adults (≥18 years)
- Treatment duration: <3 versus ≥3 years
- Years of follow up: end of treatment, 2 versus ≥2 years
- Route of administration: e.g. SCIT versus SLIT
- Allergens used for AIT
- Severity of food allergy: mild/moderate versus severe
- Primary versus secondary IgE-mediated food allergy.

Where possible, publication bias will be assessed through the creation of funnel plots, and tested by Egger’s regression test and Begg’s rank correlation test [21, 22].

### Registration and reporting

This review will be registered with the International Prospective Register of Systematic Reviews (PROSPERO): http://www.crd.york.ac.uk/prospero/. The Preferred Reporting Items for Systematic Reviews and Meta-Analyses (PRISMA) checklist will be used to guide the reporting of the systematic review: http://www.prisma-statement.org/.

## Discussion

This review will involve systematically identifying, critiquing and synthesizing the evidence on the effectiveness, cost-effectiveness and safety of AIT for the management of IgE mediated food allergy. It will build on earlier reviews in this area [10, 11]. The findings from this review will be used to inform the development of recommendations for EAACI’s Guidelines on AIT. We anticipate that this review will report in 2016.

## Additional file

10.1186/s13601-016-0113-z Appendix 1: Search strategy.
