# Erratum to: Allergen immunotherapy for IgE-mediated food allergy: protocol for a systematic review

**DOI:** 10.1186/s13601-017-0166-7

**Published:** 2017-09-15

**Authors:** Sangeeta Dhami, Ulugbek Nurmatov, Giovanni Battista Pajno, Montserrat Fernandez-Rivas, Antonella Muraro, Graham Roberts, Cezmi Akdis, Montserrat Alvaro-Lozano, Kirsten Beyer, Carsten Bindslev-Jensen, Wesley Burks, George du Toit, Motohiro Ebisawa, Philippe Eigenmann, Edward Knol, Mika Makela, Kari Christine Nadeau, Liam O’Mahony, Nikolaos Papadopoulos, Lars Poulsen, Cansin Sackesen, Hugh Sampson, Alexandra Santos, Ronald van Ree, Frans Timmermans, Aziz Sheikh

**Affiliations:** 1 Evidence-Based Health Care Ltd, Edinburgh, UK; 2 Division of Population Medicine Neuadd Meirionnydd, School of Medicine, Cardiff University, Heath Park, Cardiff, UK; 3 Allergy Unit, Department of Pediatric, University of Messina, Messina, Italy; 4 Allergy Department, Hospital Clínico San Carlos, IdISSC, Madrid, Spain; 5 Food Allergy Referral Centre Veneto Region, Department of Women and Child Health, Padua General University Hospital, Padua, Italy; 6 The David Hide Asthma and Allergy Research Centre, St Mary’s Hospital, Newport Isle of Wight, NIHR Respiratory Biomedical Research Unit, University Hospital Southampton NHS Foundation Trust, Southampton, UK; 7 Faculty of Medicine, University of Southampton, Southampton, UK; 8 Swiss Institute for Allergy and Asthma Research, Davos, Switzerland; 9 Paediatric Allergy and Clinical Immunology Section, Hospital Sant Joan de Déu, Universitat de Barcelona, Barcelona, Spain; 10 Pediatric Pneumology and Immunology, Charité Universitätsmedizin, Berlin, Germany; 11 Icahn School of Medicine at Mount Sinai, New York, NY USA; 12 Department of Dermatology and Allergy Centre, Odense University Hospital, Odense, Denmark; 13 Department of Pediatrics, School of Medicine, University of North Carolina at Chapel Hill, Chapel Hill, NC USA; 14 Division of Asthma, Allergy and Lung Biology, Department of Paediatric Allergy, MRC and Asthma Centre in Allergic Mechanisms of Asthma, King’s College London and St Thomas NHS Foundation Trust, London, UK; 15 Department of Allergy, Clinical Research Center for Allergy and Rheumatology, Sagamihara National Hospital, Sagamihara, Kanagawa Japan; 16 University Hospitals of Geneva and Medical School, University of Geneva, Geneva, Switzerland; 17 Department of Dermatology and Allergology, University Medical Center Utrecht, Utrecht, The Netherlands; 18 Department of Immunology, University Medical Center Utrecht, Utrecht, The Netherlands; 19 Skin and Allergy Hospital, Helsinki University Hospital, Helsinki, Finland; 20 Division of Immunology, Allergy and Rheumatology, Department of Pediatrics, Stanford University, Stanford, CA USA; 21 Swiss Institute of Allergy and Asthma Research (SIAF), University of Zurich, Davos, Switzerland; 22 Department of Allergy, 2nd Pediatric Clinic, University of Athens, Athens, Greece; 23 Allergy Clinic, Copenhagen University Hospital, Gentofte, Denmark; 24 Koç University Hospital, Istanbul, Turkey; 25 World Allergy Organization (WAO), Milwaukee, WI USA; 26 Division of Asthma Allergy and Lung Biology, Department of Paediatric Allergy, King’s College London/Guy’s, and St Thomas’ Hospital NHS Foundation Trust, London, UK; 27 Department of Otorhinolaryngology, Academic Medical Center, Amsterdam, The Netherlands; 28 Nederlands Anafylaxis Netwerk - European Anaphylaxis Taskforce, Dordrecht, The Netherlands; 29 Allergy and Respiratory Research Group, The University of Edinburgh, Edinburgh, UK

## Erratum to: Clin Transl Allergy (2016) 6:24 DOI 10.1186/s13601-016-0113-z

Unfortunately this article [1] was published with an error in the Funding section. The BM4SIT project is not acknowledged. This section should be corrected to the below:

**Funding**

EAACI and the BM4SIT project (Grant Number 601763) in the European Union’s Seventh Framework Programme FP7.
