# Supplementary material for: Allergen immunotherapy for IgE-mediated food allergy: protocol for a systematic review
Source: Clin Transl Allergy. 2016 Jul 5;6:24. doi: 10.1186/s13601-016-0113-z (PMC4932703; doi:10.1186/s13601-016-0113-z)
Supplement: Supplementary file 1 — 10.1186/s13601-016-0113-z Appendix 1: Search strategy. [file 13601_2016_113_MOESM1_ESM.docx]

**Appendix 1: Search strategy**

***Search strategy 1***

(MEDLINE, EMBASE, GLOBAL HEALTH, AMED and CAB)

1. exp Food Hypersensitivity/
2. exp Milk Hypersensitivity/
3. exp Egg Hypersensitivity/
4. exp Peanut Hypersensitivity/
5. exp Tree nut Hypersensitivity/
6. exp Nut Hypersensitivity/
7. ((food or Oral Allergy Syndrome or milk or egg or peanut or arachis hypogaea or tree nut or hazelnut or brazil nut or walnut or chestnut or pistachio or almond or legumes or wheat or rice or soy or fish or seafood or shellfish or shrimp or lobster or crab or crawfish or kiwi or apple or peach or apricot or cherry or pear or plum or tomato or green pea or potato or carrot or parsley or celery or additives) adj3 (allerg* or hypersensitivit*)).mp. [mp=title, original title, abstract, name of substance word, subject heading word, unique identifier]
8. or/1-7
9. exp Desensitization, Immunologic/
10. exp Immunotherapy/
11. Desensiti?ation.mp.
12. Hyposensitisation.mp.
13. Allergy vaccination.mp.
14. Immunotherapy.mp.
15. Oral Immunotherapy.mp.
16. Oral desensiti?ation.mp.
17. Specific oral tolerance induction.mp.
18. Oral tolerance induction.mp.
19. Sublingual immunotherapy.mp.
20. Epicutaneous immunotherapy.mp.
21. Specific immunotherapy.mp.
22. Or/9-21
23. exp Intervention Studies/
24. Intervention Studies.mp.
25. Experimental stud*.mp.
26. exp Clinical Trial/
27. Trial.mp.
28. Clinical Trial.mp.
29. exp Controlled Clinical Trial/
30. Controlled Clinical Trial.mp.
31. Randomi?ed Controlled Trial.mp.
32. Quasi-randomi?ed trial.mp.
33. Non-randomi?ed trial.mp.
34. exp Placebos/
35. Placebos.mp.
36. exp Random Allocation/
37. Random Allocation.mp.
38. exp Double-Blind Method/
39. Double-Blind Method.mp.
40. Double-Blind design.mp.
41. exp Single-Blind Method/
42. Single-Blind Method.mp.
43. Single-Blind design.mp.
44. Triple-Blind Method.mp.
45. Random*.mp.
46. Exp.Case series/
47. (Case$ and series).tw.
48. Cost:.mp.
49. Cost effective:.mp.
50. Cost utility:.mp.
51. Exp Health care Costs/
52. (Costs and Costs Analysis).mp.
53. Economic evaluation*.mp.
54. ((cost effective* adj1 analys*) or cost minimi?ation analys* or cost benefit analys* or cost utility analys* or cost consequence analys* or finances).mp.
55. Or/23-54
56. 8 and 22 and 55

# *Search strategy 2*

(Cochrane Library, TRIP, CINAHL, ISI Web of Science, BIOSIS)

(Food hypersensitivity or food allergy or Oral Allergy Syndrome or milk allergy or egg allergy or nut allergy or peanut allergy or arachis hypogaea allergy or tree nut allergy or hazelnut allergy or legumes allergy or wheat allergy or soy allergy or fish allergy or seafood allergy or shellfish allergy or kiwi allergy or apple allergy or peach allergy or additives hypersensitivity or additives allergy)

AND

(Immunologic, desensiti* or immunotherapy or hyposensitisation or oral immunotherapy or sublingual immunotherapy or epicutaneous immunotherapy or specific immunotherapy or oral desensiti* or Specific Oral Tolerance Induction or Oral Tolerance Induction)

AND

(Intervention stud* or experimental stud* or trial or clinical trial* or controlled clinical trial or randomi* controlled trial or random allocation or single blind method or double blind method or triple blind method or random* or case series or economic evaluation* or cost effective* analys* or cost minimization analys* or cost benefit analys* or cost utility analys* or cost consequence analys* or finances)
